# Candidate genes of the transcellular and paracellular calcium absorption pathways in the small intestine of laying hens

**DOI:** 10.3382/ps/pez407

**Published:** 2019-07-22

**Authors:** A Gloux, N Le Roy, A Brionne, E Bonin, A Juanchich, G Benzoni, M-L Piketty, D Prié, Y Nys, J Gautron, A Narcy, M J Duclos

**Affiliations:** 1 BOA, INRA, Université de Tours, 37380 Nouzilly, France; 2 GeT-PlaGe, INRA, Auzeville, 31326 Castanet-Tolosan, France; 3 Prospective and Innovation department, Neovia, 56250 Saint-Nolff, France; 4 Service des Explorations Fonctionnelles, G.H. Necker Enfants Malades, 75743 Paris Cedex 15, France, Université Paris Descartes Faculté de Médecine, INSERM U1151

**Keywords:** laying hen, Ca absorption, gene expression, transcellular pathway, paracellular pathway

## Abstract

To meet the high calcium (**Ca**) demand during eggshell biomineralization (2 g of Ca per egg), laying hens develop specific metabolic regulations to maintain Ca homeostasis. The intake of Ca, its solubilization, and absorption capacity are enhanced at sexual maturity (**SM)**. A better knowledge of the intestinal Ca transporters involved in their variations at this stage could indicate new nutritional strategies to enhance Ca digestive utilization. Transcellular Ca absorption pathway and its major player calbindin-D 28 K (*CALB1*) mediate a saturable transport, which has been extensively described in this model. Conversely, a contribution by the paracellular pathway involving non-saturable Ca transport through intercellular tight junction has also been suggested. The aim of the present study was to identify candidate genes of these two pathways and their patterns of expression, in immature pullets (12, 15, and 17 wk old) and mature laying hens (23 wk old) in the duodenum, jejunum, and ileum. Using RT-qPCR, this study identifies 3 new candidate genes for transcellular, and 9 for paracellular Ca transport. A total of 5 candidates of the transcellular pathway, transient receptor potential cation channels subfamily C member 1 (*TRPC1*) and M member 7 (*TRPM7*); *CALB1* and ATPase plasma membrane Ca^2+^ transporting 1 (*ATP2B1*) and ATPase plasma membrane Ca^2+^ transporting 2 (*ATP2B2*) were enhanced with age or after SM in the duodenum, the jejunum or all 3 segments. A total of 4 candidates of the paracellular pathway Claudin 2 (*CLDN2*) and tight junction proteins 1, 2, and 3 (*TJP1, TJP2* and *TJP3*) increased in the small intestine after SM. Additionally, *CALB1, ATP2B2*, and *CLDN2* were overexpressed in the duodenum or the jejunum or both segments after SM. The enhanced expression of candidate genes of the paracellular Ca pathway after SM, supports that the non-saturable transport could be a mechanism of great importance when high concentrations of soluble Ca are observed in the intestinal content during eggshell formation. Both pathways may work cooperatively in the duodenum and jejunum, the main sites of Ca absorption in laying hens.

## INTRODUCTION

In laying hens, eggshell formation represents a fast mineralization process, which takes place daily in the uterus, with the deposition of Calcium (**Ca**) carbonate (Nys and Le Roy, 2017). Large amounts of Ca, about 2 g per egg, must be supplied to the uterine gland, mainly during the dark period, when the hen is not consuming feed (Bar, 2009). To meet this demand, the hen adapts its daily Ca intake (4.2 to 4.6 g/d) and retention, notably by optimizing its intestinal Ca absorption during the ovulatory cycle (Hurwitz and Bar, 1965; Bar, 2009). Before the dark period, laying hens exhibit a specific appetite for Ca and crop dilatation favors enhanced acid secretion in the proventriculus, increasing Ca solubilization (Mongin and Sauveur, 1974; Sauveur and Mongin, 1983; Guinotte et al., 1995). These mechanisms facilitate the intense Ca absorption during eggshell formation (Hurwitz and Bar, 1965, 1969). Incorporation of coarse calcium carbonate into the diet can improve Ca retention by extending the supply of soluble Ca in the intestine during the dark period (Guinotte et al., 1995). When the content of soluble Ca in the intestine becomes limiting, Ca is mobilized from the medullary bone formed before the onset of laying which constitutes a labile source of Ca (Castillo et al., 1979; Sauveur and Mongin, 1983; Kerschnitzki et al., 2014). Any imbalance in Ca homeostasis impairs the quality of the eggshell or bone mineralization and strength, and Ca deficiency is the primary cause of osteoporosis. It coincides with a progressive loss of mineralized structural bone which leads to bone fragility and susceptibility to fracture (Whitehead, 2004). It represents a common problem in laying flocks causing both financial losses and welfare issues which can occur as soon as 45 wk old (Kim et al., 2012; Olgun and Aygun, 2016). Many dietary factors interacting with Ca digestibility and retention have already been studied, including phytic acid content (Jalal and Scheideler, 2001; Lim et al., 2003), Ca to Phosphorus (**P**) ratio (Keshavarz, 1986; Lim et al., 2003) and vitamin D_3_ levels (Plaimast et al., 2015). An improved knowledge about mechanisms involved in intestinal Ca absorption in laying hens could help to define new nutritional strategies to enhance its digestive utilization. In laying hens, Ca absorption occurs in the small intestine, and increases mainly in the duodenum and jejunum during eggshell formation, due to high Ca absorption rates (Hurwitz and Bar, 1965; Hurwitz and Bar, 1968). Both active (saturable) and passive (non-saturable) Ca transport have been reported during the eggshell formation period (Hurwitz and Bar, 1969; Nys and Mongin, 1982). Active Ca transport, mediated by the transcellular Ca absorption pathway, has been intensively studied and several of its components have been described, in contrast to the mechanisms of passive Ca transport. The major player in the transcellular pathway is the vitamin D_3_-induced Ca-binding protein, named calbindin-D 28K (***CALB1***), initially discovered in the chick's intestine (Wasserman and Taylor, 1966). Other members of the transcellular pathway, in charge of enterocyte Ca^2+^ entry Transient receptor potential cation channel subfamily V member 6 (***TRPV6***) and extrusion ATPase plasma membrane Ca^2+^ transporting 1 (***ATP2B1***), ATPase plasma membrane Ca^2+^ transporting 2 (***ATP2B2***), ATPase plasma membrane Ca^2+^ transporting 4 (***ATP2B4***), and Solute Carrier Family 8 Member A1 (*SLC8A1*) have also been detected in the duodenum of laying hens (Yang et al., 2011; Jonchère et al., 2012). However, a complete description of these candidates is lacking in the different segments of the small intestine of the laying hen.

Controversies have also been raised about *TRPV6* expression in the small intestine of laying hens or chickens, suggesting that other candidates should be investigated (Yang et al., 2011; Proszkowiec-Weglarz et al., 2019).

Duodenal CALB1 concentration correlates positively to the percentage of net Ca absorption, but negatively to the daily amount of Ca absorbed (Bar et al., 1979). In addition, no variation of duodenal CALB1 concentration occurs during the ovulatory cycle (Nys et al., 1992). These observations support the hypothesis that a non-saturable transport can contribute to Ca absorption in laying hens having high Ca intake.

In mammalian species, passive Ca transport relies on the paracellular pathway. It consists of intercellular tight junctions (**TJ**) composed of transmembrane (claudin: **CLDN**, junctional adhesion molecule**: JAM** and occludin**: OCLN)** and cytosolic (tight junction proteins: **TJP**), proteins which form pores for the permeation of ions and water. The nature of the CLDN determines the specificity of pores for Ca transport and overexpression of Claudin 2 (***CLDN2***) or *CLDN12* enhances Ca transfer through intestinal epithelial cells in vitro (Fujita et al., 2008). Intestinal Ca transport is mediated by the paracellular pathway when dietary Ca is high and by the transcellular pathway when dietary Ca is low (Christakos et al., 2014). Moreover both pathways may cooperate, as invalidation of Calbindin-D 9K in mice induces an overexpression of *CLDN2* and *15* (Hwang et al., 2013). The paracellular pathway has not been studied so far in the laying hens. If present and functional it could participate in the intestinal Ca transport, when they exhibit high Ca intake to match the high Ca demand for eggshell mineralization. As a first step to support this hypothesis, the present study identifies candidate genes encoding transmembrane and cytosolic proteins of the TJ in the small intestine of laying hens. In the domestic hen, sexual maturity (**SM**) occurs with the onset of the reproductive function and the first oviposition (García-Fernández et al., 2015). This transition is characterized by a huge increase in intestinal Ca absorption capacity (up to 6-fold), and a rise of 1.25(OH)_2_D_3_ plasma levels (Castillo et al., 1979; Nys et al., 1986b, 1992). Duodenal *CALB1* mRNA and protein levels increase at SM (Nys et al., 1986b, 1992). We hypothesize that genes with an increased expression profile between immature and mature hens would be strong candidates to mediate Ca absorption.

The objective of the present study was therefore to identify candidate genes of the transcellular and paracellular pathways and their patterns of expression in immature pullets (12, 15, and 17 wk of age) and mature laying hens (23 wk of age), in the 3 segments of the small intestine duodenum, jejunum and ileum.

## MATERIALS AND METHODS

### Animals

The experiment was conducted under the guidelines of the French Ministry of Agriculture for Animal Research at the Experimental Poultry Unit of Tours, INRA, Nouzilly, France (PEAT INRA 1295). The experimental design was approved by the Regional Ethics Committee on animal experimentation (Tours, France) and the French Ministry of Higher Education and Research (Paris, France; authorization: 10043). Animals had free access to water and feed, with diets adapted to their physiological status (Table 1). Thirty 10-wk-old pullets (Isa Brown strain, Hendrix Genetics Layers, Ploufragan, France) were raised collectively in 3 pens (10 hens per per) until their slaughter at 12, 15, and 17 wk old, following the breeder guidelines for diet and environmental conditions (Hendrix Genetics, Ploufragan, France).

**Table 1.** Diet composition for pullets (from 12 to 17 wk old) and laying hens (from 20 to 23 wk old).

	Diet	
Item	Pullet	Laying hen
Ingredient, %		
Corn	47.0	35.5
Wheat	25	25
Soybean meal	18.8	22.7
Wheat bran	6	5
Soybean oil	0.3	1.43
Corn gluten meal	1.00	
Limestone CaCo3	1.2	5.07
Ground CaCO3	2.53	
Dicalcium phosphate	0.8	0.7
NaCl	0.24	0.29
Sodium bicarbonate	0.18	0.07
Premix	0.5*	0.5^†^
DL-methionine	0.07	0.15
L-Thr		0.01
Calculated energy and nutrient composition, as-fed		
ME, kcal/kg	2,886	2,711
CP, %	16.0	17.3
Ca, %	1.0 (0.99)	3.5 (3.9)
Total P, %	0.53 (0.54)	0.50 (0.48)
Available P, %	0.4	0.3
Fat, %	3.1	3.8
Cellulose, %	3.0	2.9
Ash, %	5.2	11.5

*Pullet's premix contains the following per kilogram: vitamin A = 10,000 IU; vitamin D_3_ = 2,500 IU; vitamin E = 30 IU; vitamin K = 2 mg; vitamin B_1_ = 2 mg; vitamin B_2_ = 6 mg; nicotinic acid = 40 mg; pantothenic acid = 10 mg; vitamin B_6_ = 3 mg; folic acid = 0.6 mg; vitamin B_12_ = 0.013 mg; biotin = 0.1 mg; Fe = 50 mg; Zn = 75 mg; Mn = 80 mg; Cu = 15 mg; I = 1.5 mg; and Se = 0.3 mg.

† Laying hen's premix contains the following per kilogram: vitamin A = 8,000 IU; vitamin D_3 =_ 2,000 IU; vitamin E = 20 IU; vitamin K = 1 mg; vitamin B_1_ = 2 mg; vitamin B_2_ = 4 mg; nicotinic acid = 8 mg; pantothenic acid = 20 mg; vitamin B_6_ = 3 mg; vitamin B_12_ = 0.005 mg; Fe = 18 mg; Zn = 73 mg; Mn = 90 mg; Cu = 5 mg; I = 1 mg; and Se = 0.15 mg.

(): analyzed nutritional values.

Ten mature 20-wk-old laying hens from the same strain were placed in individual cages until 23 wk old, which represents the beginning of the peak of lay. They were subjected to 14L:10D light program. The hens were euthanized 9 to 10 h after ovulation, as measured by regular monitoring of oviposition (every 10 min), and the precise stage in the ovulatory cycle was confirmed based on the weight of the dried eggshell, as previously described (Nys et al., 2010).

### Tissue Sampling

Six pullets per age at 12, 15, 17 wk old, and 7 laying hens at 23 wk old at the beginning of eggshell formation (9 to 10 h post-ovulation) were submitted to blood collection in the occipital sinus using heparin–lithium coated tubes. They were then euthanized by an intravenous injection of Dolethal (182 mg/ml, pentobarbital sodium, Vetoquinol, France). Blood was centrifuged immediately at 1000 g x 15 min for plasma collection and storage at −20°C for further analysis. The oviduct was removed and weighed for immature pullets. For each hen the duodenum, jejunum, and ileum were immediately removed and thoroughly washed with a cold PBS solution. A 4-cm sample was isolated at the rostral part of the respective segments: the duodenum, the jejunum, the ileum, scraped and snap-frozen into liquid nitrogen, prior to storage at −80°C until RNA extraction.

### Blood Sample Analysis

Total plasma Ca and P concentrations were measured using inductively coupled plasma optical emission spectrometry (ICP OES ThermoscientificTM iCAPTM 7200; method 990.08; AOAC International, 2006). Determination of 25(OH)D_3_ and 1.25(OH)_2_D_3_ concentrations were performed by chemiluminescent immunoassay (Diasorin, Saluggia, Italy, Spanaus and von Eckardstein, 2017) at the Liaison XL platform Service des Explorations Fonctionnelles (G.H. Necker Enfants Malades, 75743, Paris cedex 15, France).

### Selection of Candidate Genes and Primer Design

Candidate genes were selected according to their identification in previous studies on mammals or chickens (Fujita et al., 2008; Yang et al., 2011; Jonchère et al., 2012; Gunzel and Yu, 2013; Hwang et al., 2013; Rousseau et al., 2016). For genes identified from mammals, the retrieval of the orthologous sequence was performed in Gallus_gallus-5.0 version of the chicken genome assembly using Ensembl 91 (December 2017).

Analysis of published RNAseq data on chicken gastro-intestinal tracts (Juanchich et al., 2018) allowed us to check the presence of genes previously selected from the literature and provided additional candidates. Primers were then designed with the aid of Primer-BLAST (Ye et al., 2012). The validation of primers was performed by RT-qPCR, using a LightCycler 480 Instrument II (Roche Applied Science, Meylan, France), considering primer efficiency between 80 and 120%, and sequencing of the PCR product (Genewiz, United Kingdom). We obtained a final list of 20 candidate genes and 7 housekeeping genes (**HKG**) with their validated primers (Tables 2, 3 and 4). All genes were named and abbreviated according to the CNGC Chicken Nomenclature genome Consortium (http://birdgenenames.org/cgnc).

**Table 2.** PCR primers for candidate genes of the transcellular pathway.

Gene symbol	Gene name	Function/location*	Forward/reverse primer	Amplicon size (bp)	Accession #
TRPV2	Transient receptor potential cation channel subfamily V member 2	cation channel/APM	ACTTCCCCTCTCTTTGGCTG/AGTCTTCACACCTGCCTTCA	211	XM_004946685.3
TRPC1	Transient receptor potential cation channel subfamily C member 1	cation channel/APM	CATCGAGTGGCAAAGTGAAA/AGTTCGAAAGCCAAGGAGGT	233	NM_001004409.2
TRPM7	Transient receptor potential cation channel subfamily M member 7	cation channel/APM	GTGTTCCCAGGAAGGCAATA/GCTTGAAGAAATGGGGTCAA	196	NM_001177555.1
CALM1	Calmodulin	control ion channel/C	GGTCGCTGGGTCAAAATCC/ACTCGGAATGCCTCACGGA	163	NM_001110364.1
CALB1^†^	Calbindin 28K	intracellular Ca^2+^ binding protein/C	CAGGGTGTCAAAATGTGTGC/GCCAGTTCTGCTCGGTAAAG	215	NM_205513.1
ATP2B1^†^	ATPase plasma membrane Ca^2+^ transporting 1	Ca^2+^ /H^+^ exchange pump/BPM	CTGCACTGAAGAAAGCAGATGTTG/GCTGTCATATACGTTTCGTCCCC	146	NM_001168002.3
ATP2B2^†^	ATPase plasma membrane Ca^2+^ transporting 2	Ca^2+^ /H^+^ exchange pump/BPM	TTACTGTACTTGTGGTTGCTGTCCC/GGTTGTTAGCGTCCCTGTTTTG	176	XM_025154762.1
ATP2B4^†^	ATPase plasma membrane Ca^2+^ transporting 4	Ca^2+^/H^+^ exchange pump/BPM	TGCTCTGAAGAAAGCTGATGTTGG/GCTGGTGAAGTTGTCATCCGTC	103	XM_015298964.2

* APM = apical plasma membrane, C = cytosol, BPM = basal plasma membrane.

† Primer sequence designed by Jonchère et al. (2012).

**Table 3.** PCR primers for candidate genes of the paracellular pathway.

Gene symbol	Gene name	Function/location*	Forward/reverse primer	Amplicon size (bp)	Accession #
CLDN2	Claudin 2	increase permeability to Ca^2+^/AIS	CGCTCGTATCTCTTGCTTGG/AGAGTATGGCTGTGACGAGG	185	NM_001277622.1
CLDN12	Claudin 12	increase permeability to Ca^2+^/AIS	ACGAGAGGAATGTGACCGTT/TTGGCACGCTTGATACGAAG	225	XM_025148431.1
CLDN1	Claudin 1	decrease permeability to cations/AIS	AGATCCAGTGCAAGGTGTACG/CTGACAGACCTGCAATGATGAAG	216	NM_001013611.2
CLDN10	Claudin 10	increase permeability to cations/AIS	TCCAACTGCAAGGACTTCCC/GCACAGCCCACACAGTATGA	210	NM_001277767.1
TJP1	Tight junction protein 1	tight junction protein connecting transmembrane proteins/C	ACCGAGAGATGCTGGTACTG/GCACAGCCTCATTCTCATGG	208	XM_015278981.2
TJP2	Tight junction protein 2	tight junction protein connecting transmembrane proteins/C	CATTGTTCGGGAGGATGCTG/AGCCAGCCAGTTTCCTAGTT	247	NM_204918.1
TJP3	Tight junction protein 3	tight junction protein connecting transmembrane proteins/C	GGATACAGTGCGGCAGATTG/TGGTAGCAGTGAAGAGGTGG	245	XM_015299758.2
OCLN	Occludin	barrier protein of tight junction/AIS and C	CCGTAACCCCGAGTTGGAT/ATTGAGGCGGTCGTTGATG	214	NM_205128.1
JAM2	Junctional adhesion molecule 2	barrier protein of tight junction/AIS and C	AGACAGGAACAGGCAGTGCT/TCCAATCCCATTTGAGGCTA	134	NM_001006257.1

* AIS = apical intercellular space, C = cytosol.

**Table 4.** PCR primers for vitamin D receptor, phosphorus transporters, and housekeeping genes.

Gene symbol	Gene name	Function/location*	Forward/reverse primer	Amplicon size (bp)	Accession #
VDR	Vitamin D3 receptor	Vitamin D_3_ receptor/N	CGATGTTCACCTGTCCGTTC/CGATGACTTTCTGCTGCTCC	231	NM_205098.1
SLC20A1^†^	Solute carrier family 20 member 1	Sodium-phosphate symporter/APM	AGGGCAGAAAGGCGTCAA/CGAGGAAGAAGAGAACAGCAGA	104	XM_015297502.2
SLC34A2^†^	Solute carrier family 34 member 2	Sodium-phosphate symporter/APM	GTCCGTTCACTCTGTTGCCT/TGGGTCCTCTTCTTGCCTTG	242	NM_204474.2
EIF3I	eukaryotic translation initiation factor 3 subunit I	Housekeeping gene/C	GACATGTGCTCACTGGCTCT/CACTGCTGAGCTGGTCTTCA	95	NM_001164395.1
EIF3F	eukaryotic translation initiation factor 3 subunit F	Housekeeping gene/C	CTAACTGCTTCTCCGTCCCG/ATGTCGTGCCCTGTTGCATA	142	XM_421624.4
GAPDH	glyceraldehyde-3-phosphate dehydrogenase	Housekeeping gene/C and N	TCTCTGTTGTTGACCTGACCTG/ATGGCTGTCACCATTGAAGTC	155	NM_204305.1
PPIA	peptidylprolyl isomerase A	Housekeeping gene/C	CGCTGACAAGGTGCCCATAA/GTCACCACCCTGACACATGA	124	NM_001166326.1
SDHA	succinate dehydrogenase complex flavoprotein subunit A	Housekeeping gene/C	AGATACGGGAAGGAAGGGGT/ACCGTAGGCAAAACGGGAAT	169	NM_001277398.1
TBP	TATA-box binding protein	Housekeeping gene/N	GCGTTTTGCTGCTGTTATTATGAG/TCCTTGCTGCCAGTCTGGAC	122	NM_205103.1
MATR3	matrin 3	Housekeeping gene/N	ATTCACAAGGTCATGGGCGT/CCTTCCAAGAGATGCTGGCA	92	NM_204147.1

* APM = apical plasma membrane, C = cytosol, N = nucleus.

† Primer sequence designed by Rousseau et al. (2016).

### RNA Extraction and Reverse Transcription

Total RNA was extracted using the method of Chomczynski and Sacchi, (1987) according to the manufacturer's recommendations (RNANow, Ozyme, Saint-Quentin en Yvelines, France). Concentration and quality of the extracted RNA were assessed by spectrophotometry from 230 to 280 nm, using a Nanodrop 1000 spectrophotometer (Nanodrop Technology, Wilmington, USA). The ratios 260/280 and 260/230 were between 1.8 and 2.2. The integrity of RNA was assessed by the migration of total RNA on a 1.5% agarose gel. Total RNA samples (5 μg) were subjected to DNase (DNAfree, Invitrogen) and then reverse transcripted using RNase H-MMLV reverse transcriptase (Superscript II, Invitrogen, Cergy Pontoise, France) and random hexamers (Amersham, Orsay, France).

### Quantitative RT-PCR

High throughput real-time quantitative PCR was performed using the Biomark microfluidic system from Fluidigm, in which every sample-gene combination is quantified using 96.96 Dynamic Array IFCs (BMK-M-96.96, Fluidigm). Pre-amplification of the samples, chip loading and qRT-PCR were performed according to the manufacturer's protocol. Results were analyzed using the real-time PCR analysis software v.4.1.3 (Fluidigm).

### Calculation of Relative Gene Expression

The relative mRNA quantification was performed with the ΔΔCt calculation method proposed by Pfaffl (2001). The relative expression ratio (*R*) of a candidate gene was calculated, based on the Efficiency (E) and the cycle threshold (Ct) deviation of a cDNA sample (the duodenum, jejunum, and ileum of individuals) vs. a control (mix of all cDNA samples at similar concentration), and expressed in comparison to the geometric average of a set of HKG according to the following equation of Pfaffl (2001).

$$\begin{eqnarray*}R &=& {\left( {1 + {\rm{E}}\,\,{\rm{candidate}}} \right)^{\Delta{\rm{Ct}}\,\,{\rm{candidate}}\,\,({\rm{control}}-{\rm{sample}}}})/\nonumber \\
&& {\rm{Geometric}}\,\,{\rm{Average }}\left[ {{{\left( {1 + {\rm{E}}\,\,{\rm{HKG}}} \right)}^{\Delta{\rm{Ct}}\,\,{\rm{HKG}}\,\,({\rm{control}}-{\rm{sample}})}}}\right ]\end{eqnarray*}$$

The 7 most stable HKG (out of 11) were chosen using GeNorm (Vandesompele et al., 2002).To facilitate the reading of the data tables and figures, the relative gene expressions were multiplied by 100.

### Statistical Analyses

All statistical analyses were performed using R 3.4.0 software (R Core Team, 2017, Vienna, Austria). For plasma levels of total Ca, P, and vitamin D metabolites, the assumptions of the linear model were validated, and the data analyzed by one-way ANOVA, including the hen age as the main effect. Significant differences between means (*P* < 0.05) were further separated using a Tukey's test.

For mRNA expression, the normality of the data was checked by a quantile-quantile plot. Outliers, identified on the linear relationship between theoretical and sample percentiles, were removed from the dataset, resulting in a slight reduction in the number of samples for some genes in their experimental group. Changes in mRNA expression of candidate genes were analyzed with a linear mixed model (lme function) including the interaction between age and intestinal segment. The hen, considered as the experimental unit, was included as a random effect in the model. The interaction was removed from the model when it did not significantly affect mRNA expression. When significant differences were observed (*P* < 0.05), all pairwise comparisons were performed using the least-square means (**LSMeans**) method (function emmeans) and a Tukey adjustment.

## RESULTS

### Plasma Levels of Total Ca, P, and Vitamin D Metabolites of Immature and Mature Hens

The effect of age on plasma levels of total Ca, P, and vitamin D metabolites are shown in Table 5. All recorded plasma parameters varied between 12 and 17 wk old or between 17 and 23 wk old, after SM. plasma levels of total Ca remained stable until 17 wk old, then increased markedly from 88 to 158 mg/l (*P* < 0.01) between 17 and 23 wk old. This corresponds to the transition between immature and mature stages and to the increase of dietary Ca levels. Plasma levels of total P and 25(OH)D_3_ showed similar profiles, decreasing between 12 and 17 wk old, then remaining stable between 17 and 23 wk old (*P* < 0.001 and *P* < 0.01). Plasma levels of 1.25(OH)_2_D_3_ rose gradually between 12 (40 pg/ml) and 17 wk old (140 pg/ml) and sharply between 17 and 23 wk old (400 pg/ml; *P* < 0.001). Sexual development of pullets was attested by a 10-fold increase of the oviduct's weight between 15 and 17 wk old (data not shown).

**Table 5.** Plasma levels of total calcium (Ca), phosphorus (P), and vitamin D metabolites in hens from 12 to 23 wk old.

	Age of hens (wk)				
Plasma levels	12	15	17	23	*P*-value
Total Ca (mg/dL)	703 ± 34^a^	696 ± 34^a^	884 ± 106^a^	1575 ± 70^b^	0.007
Total P (mg/dL)	420 ± 16^b^	343 ± 29^a,b^	302 ± 22^a^	296 ± 34^a^	<.0001
25(OH)D_3_ (ng/mL)	70.5 ± 5.9^b^	52.2 ± 4.8^a,b^	40.0 ± 4.9^a^	42.6 ± 4.8^a^	0.002
1.25(OH)_2_D_3_ (pg/mL)	39.3 ± 2.0^a^	79.5 ± 12.7^a,b^	140.0 ± 27.2^b^	398.9 ± 19.3^c^	<.0001

Values are presented as LSMeans ± SEM (n = 6 to 7 per experimental group).

c–a Means with different superscripts within the same column are significantly different (*P* < 0.05).

### Candidate Genes of the Transcellular Pathway

The interaction between age and intestinal segment on mRNA expression of genes of the transcellular pathway is presented in Table 6. The mRNA expression of 6 candidate genes out of 8 was affected by the interaction between age and segment: transient receptor potential cation channel subfamily V member 2 (***TRPV2***) (*P* < 0.01), transient receptor potential cation channels subfamily M member 7 (***TRPM7***) (*P* < 0.05), calmodulin *(CALM1*) (*P* < 0.01), *CALB1* (*P* < 0.001), *ATP2B2* (*P* < 0.001), and *ATP2B4* (*P* < 0.001). The mRNA expression of *TRPV2* was higher in the jejunum at 12 and in the ileum at 17 wk old than in the duodenum at 12 wk old. It was then similar across segments from 15 to 23 wk old. Gene expression of *TRPM7* increased in the jejunum between 12 and 23 wk old, and was similar between segments at 23 wk old, after SM. The mRNA expression of *CALM1* was similar across segments at each age, but was higher in the duodenum at 23 than in the jejunum at 12 wk old. The mRNA expressions of *CALB1* and *ATP2B2* were similar across ages and segments until 17 wk old, but rose sharply between 17 and 23 wk old in the duodenum and jejunum for *CALB1*, and in the duodenum for *ATP2B2*. At 23 wk old, the expression of *CALB1* was similar in the duodenum and jejunum and higher than in the ileum, and the expression of *ATP2B2* was higher in the duodenum than in the other segments. The mRNA expression of *ATP2B4*, which was highest in the jejunum at 12 wk old, decreased in this segment between 12 and 15 wk old, while it increased in the ileum. The mRNA expression of *ATP2B4* at 23 wk old was higher in the ileum than in the other segments.

**Table 6.** Effect of interaction between age and intestinal segment on the relative mRNA expression of candidate genes of the transcellular pathway.

Gene		TRPV2	TRPM7	CALM1	CALB1	ATP2B2	ATP2B4
Age (wk)	Segment						
12	Duodenum	42^b^	61^a–c^	71^a,b^	16^b^	46^b^	19^e^
		(16)	(8)	(6)	(11)	(38)	(6)
	Jejunum	118^a^	38^c^	65^b^	1^b^	64^b^	104^a^
		(15)	(8)	(6)	(11)	(38)	(6)
	Ileum	54^a,b^	56^a–c^	92^a,b^	1^b^	39^b^	51^c–e^
		(15)	(8)	(6)	(11)	(38)	(6)
15	Duodenum	92^a,b^	101^a^	81^a,b^	15^b^	94^b^	29^c–e^
		(16)	(9)	(6)	(11)	(41)	(6)
	Jejunum	53^a,b^	68^a–c^	78^a,b^	3^b^	35^b^	58^c,d^
		(15)	(8)	(6)	(11)	(38)	(6)
	Ileum	95^a,b^	85^a,b^	74^a,b^	1^b^	87^b^	98^a,b^
		(15)	(8)	(6)	(11)	(38)	(6)
17	Duodenum	65^a,b^	89^a,b^	82^a,b^	43^b^	123^b^	25^d,e^
		(16)	(8)	(6)	(11)	(38)	(6)
	Jejunum	52^a,b^	60^a–c^	83^a,b^	13^b^	53^b^	46^c–f^
		(15)	(8)	(6)	(11)	(38)	(6)
	Ileum	110^a^	53^b,c^	63^b^	5^b^	83^b^	71^b,c^
		(15)	(8)	(6)	(11)	(38)	(6)
23	Duodenum	74^a,b^	88^a,b^	96^a^	143^a^	381^a^	31^c–e^
		(14)	(8)	(5)	(10)	(35)	(5)
	Jejunum	70^a,b^	86^a,b^	90^a,b^	109^a^	53^b^	54^c–e^
		(14)	(8)	(5)	(11)	(35)	(5)
	Ileum	61^a,b^	57^a–c^	85^a,b^	47^b^	75^b^	91^a,b^
		(14)	(8)	(5)	(10)	(35)	(5)
Source of variation							
	Age	0.860	0.002	0.011	<.0001	0.005	0.020
	Segment	0.599	0.000	0.548	<.0001	<.0001	<.0001
	Age x Segment	0.001	0.038	0.006	0.000	0.000	<.0001

Relative expression values, expressed as arbitrary units are presented as LSMeans with (SEM) with n = 5 to 7 per experimental group.

a–f Means with different superscripts within the same column are significantly different (*P* < 0.05).

*TRPV2* = Transient receptor potential cation channel subfamily V member 2; *TRPM7* = Transient receptor potential cation channel subfamily M member 7; *CALM1* = Calmodulin; *CALB1* = Calbindin 28K; *ATP2B2* = ATPase plasma membrane Ca^2+^ transporting 2; and *ATP2B4* = ATPase plasma membrane Ca^2+^ transporting 4.

**Figure 1.**
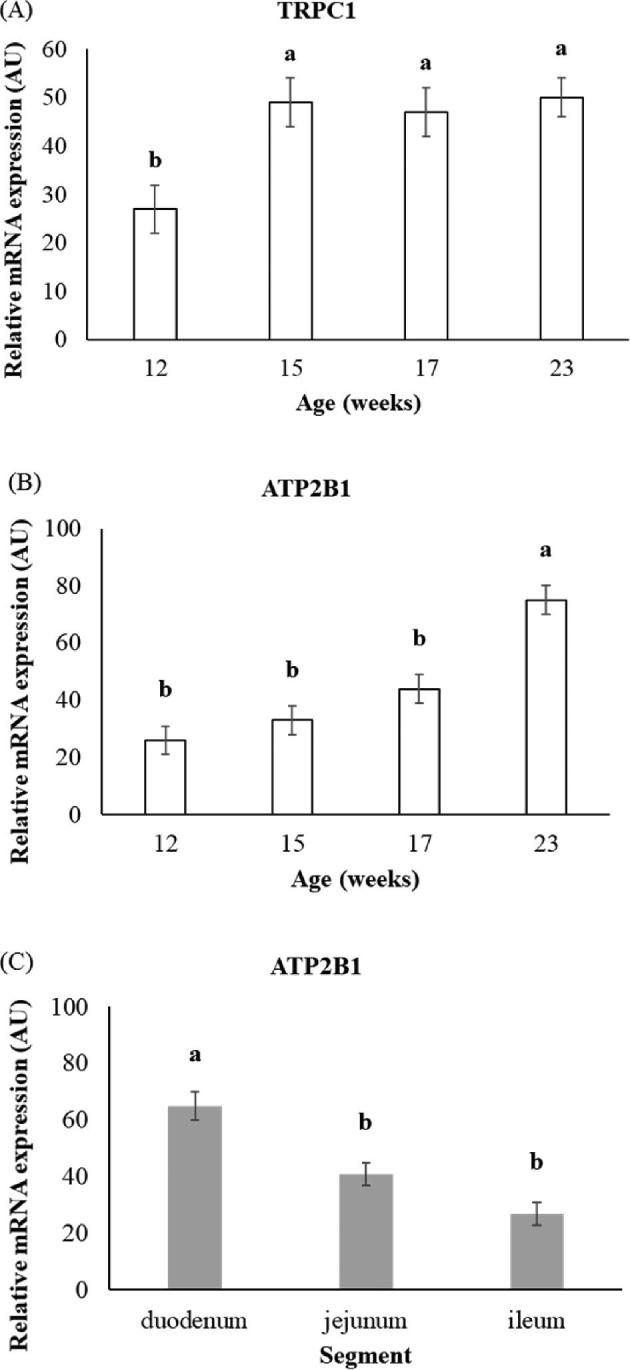
Effect of age (A-B) or segment (C) on the relative mRNA expression of candidate genes of the transcellular pathway. (A) Transient receptor potential cation channel subfamily C member 1 (*TRPC1*), (B, C) ATPase plasma membrane Ca^2+^ transporting 1 (*ATP2B1*). Values expressed as arbitrary units (AU) are LSMeans ± SEM (n = 17 to 21 per age and n = 23 to 25 per segment). ^a-b^Means with different superscripts within the graph are significantly different (*P* < 0.05).

For the remaining three candidates of the transcellular pathway, no interaction between age and segment was observed. The main effects of age and intestinal segment are presented in Figure 1. The mRNA expression of transient receptor potential cation channels subfamily C member 1 (***TRPC1***) increased between 12 and 15 wk old (*P* < 0.05), and then remained elevated (Figure 1A). The mRNA expression of *ATP2B1* rose between 17 and 23 wk old (*P* < 0.05), and its expression was higher in the duodenum compared to the other segments (*P* < 0.05; Figure 1B and 1C).

### Candidate Genes of the Paracellular Pathway

The interaction between age and intestinal segment on mRNA expression of genes of the paracellular pathway is presented in Table 7. The mRNA expression of 4 candidate genes out of 9 was affected by the interaction between age and segment: *CLDN2* (*P* < 0.01), *CLDN10* (*P* < 0.001), *OCLN* (*P* < 0.05) and *JAM2* (*P* < 0.001). The mRNA expression of *CLDN2* increased in the duodenum between 12 and 23 wk old, while it was low in the jejunum until 17 wk old, and increased between 17 and 23 wk old. As a result, the expression of CLDN2 was higher in the jejunum than in the ileum at 23 wk old. Gene expression of *CLDN10, OCLN*, and *JAM2* decreased with age in 1 or 2 segments. The mRNA expression of *CLDN10* decreased in the duodenum between 12 and 17 wk old. In the jejunum, it decreased between 17 and 23 wk old and in the ileum between 12 and 23 wk old. As a result, *CLDN10* was expressed at similar levels in the 3 segments after SM. The mRNA expression of *OCLN* was similar across the 3 segments, except at 15 wk old, when it was higher in the ileum than in the other segments. Gene expression of *JAM2* was higher in the ileum than in the other 2 segments at 15 and 17 wk old, and subsequently decreased in the ileum. As a result similar mRNA expression between segments was observed after SM.

**Table 7.** Effect of interaction between age and intestinal segment on the relative mRNA expression of candidate genes of the paracellular pathway.

Gene		CLDN2	CLDN10	OCLN	JAM2
Age (wk)	Segment				
12	Duodenum	17^c–h^	339^a^	72^b,c^	83^b,c^
		(11)	(21)	(9)	(243)
	Jejunum	5^g,h^	181^b,c^	99^b,c^	884^a–c^
		(11)	(21)	(9)	(142)
	Ileum	9^e–h^	250^a,b^	105^a–c^	435^a–c^
		(11)	(21)	(9)	(155)
15	Duodenum	53^a–g^	235^a,b^	67^c^	421^b,c^
		(11)	(23)	(10)	(155)
	Jejunum	38^b–h^	305^a^	87^b,c^	240^c^
		(11)	(21)	(9)	(142)
	Ileum	14^d–h^	179^b,c^	149^a^	1174^a^
		(11)	(21)	(9)	(142)
17	Duodenum	68^a–d^	172^b,c^	82^b,c^	355^c^
		(11)	(21)	(9)	(142)
	Jejunum	42^b–h^	237^a,b^	76^b,c^	334^c^
		(11)	(21)	(9)	(142)
	Ileum	28^e–h^	147^b–d^	119^a,b^	1078^a,b^
		(11)	(21)	(9)	(142)
23	Duodenum	87^a,b^	50^d^	87^b,c^	228^c^
		(10)	(20)	(9)	(131)
	Jejunum	99^a^	108^c,d^	85^b,c^	160^c^
		(10)	(20)	(9)	(141)
	Ileum	59^b–f^	113^c,d^	110^a–c^	357^c^
		(10)	(20)	(9)	(131)
Source of variation					
	Age	0.000	<.0001	0.462	0.024
	Segment	<.0001	0.047	<.0001	0.000
	Age x Segment	0.005	<.0001	0.015	0.000

Relative expression values, expressed as arbitrary units, are presented as LSMeans with (SEM) with n = 4 to 7 per experimental group.

a–h Means with different superscripts within the same column are significantly different (*P* < 0.05).

*CLDN2* = Claudin 2; *CLDN10* = Claudin 10; *OCLN* = Occludin; and *JAM2* = Junctional adhesion molecule 2.

**Figure 2.**
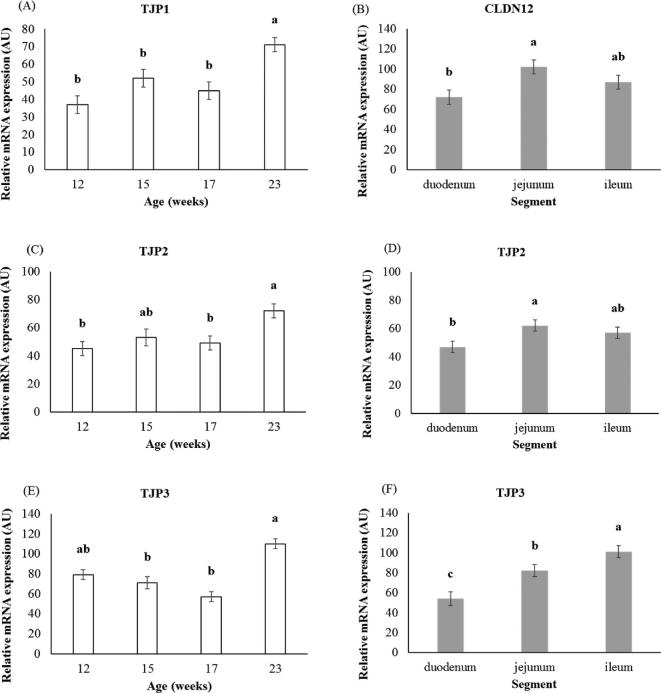
Effect of age (A-C-E) or segment (B-D-F) on the relative mRNA expression of candidate genes of the paracellular pathway (A, C) Tight junction protein 1 (*TJP1*), (B) Claudin 12 (CLDN12), (C; D) Tight junction protein 2 (*TJP2*), (E, F) Tight junction protein 3 (*TJP3*). Values expressed as arbitrary units (AU) are LSMeans ± SEM (n = 17 to 21 per age and n = 24 to 25 per segment). ^a-c^Means with different superscripts within the graph are significantly different (*P* < 0.05).

For the remaining 5 candidates of the paracellular pathway, no interaction between age and segment was observed. The effects of age and intestinal segment are shown in Figure 2. While *CLDN1* expression neither varied with age, nor with segment (data not shown), *CLDN12* expression varied with the segment (*P* < 0.05), tight junction proteins 1 (***TJP1***) expression with age, tight junction proteins 2 (***TJP2***), and tight junction proteins 3 (***TJP3***) expression with both age and segment (*P* < 0.05). The mRNA expression of *CLDN12* was higher in the jejunum than in the duodenum, but intermediate in the ileum (Figure 2B). Gene expression of *TJP1, TJP2*, and *TJP3* increased between 17 and 23 wk old (Figure 2A, 2C and 2E). The mRNA expression of *TJP2* was higher in the jejunum than in the duodenum, but intermediate in the ileum (Figure 2D). Gene expression of *TJP3* increased from the duodenum to the ileum (Figure 2F).

### Vitamin D Receptor and P Transporters

The interactions between age and intestinal segment on mRNA expression of vitamin D receptor (***VDR***) and P transporters are shown in Table 8. The mRNA expression of *VDR* increased in the jejunum between 12 and 23 wk old (*P* < 0.01), but was not affected by age in the other segments. In addition, it was similar for segments at each of the four ages. The mRNA expression of solute carrier family 20 member 1 (*SLC20A1*) was higher in the jejunum than in the duodenum between 12 and 17 wk old, and higher in the ileum than in the duodenum at 23 wk old, following a significant decrease in the jejunum between 15 and 23 wk old (*P* < 0.001). Gene expression of solute carrier family 34 member 2 (***SLC34A2***) was higher in the duodenum than in the jejunum and ileum, until 17 wk old. It subsequently increased in the jejunum between 17 and 23 wk old. Consequently its expression was similar in the duodenum and jejunum at 23 wk old, and was higher than in the ileum (*P* < 0.001).

**Table 8.** Effect of interaction between age and intestinal segment on the relative mRNA expression of candidate genes for vitamin D receptor and transcellular phosphorus apical entry.

Gene		VDR	SLC20A1	SLC34A2
Age (wk)	Segment			
12	Duodenum	78^a–d^	72^d–f^	357^a^
		(13)	(24)	(30)
	Jejunum	23^d^	189^a–c^	1^c^
		(13)	(24)	(30)
	Ileum	56^b–d^	268^a,b^	59^c^
		(13)	(26)	(30)
15	Duodenum	96^b–d^	70^c–f^	257^a,b^
		(14)	(26)	(33)
	Jejunum	102^a,b^	288^a^	93^c^
		(13)	(24)	(30)
	Ileum	50^b–d^	162^b–d^	2^c^
		(13)	(24)	(30)
17	Duodenum	69^a–d^	34^e,f^	329^a^
		(13)	(24)	(30)
	Jejunum	74^a–d^	182^a–d^	108^b,c^
		(13)	(24)	(30)
	Ileum	31^c,d^	115^c–f^	3^c^
		(13)	(24)	(30)
23	Duodenum	103^a,b^	24^f^	315^a^
		(12)	(22)	(28)
	Jejunum	119^a^	110^c–f^	265^a^
		(12)	(22)	(28)
	Ileum	86^a–d^	152^b–e^	54^c^
		(12)	(22)	(28)
Source of variation	*P*-value			
	Age	0.001	0.003	0.003
	Segment	0.001	<.0001	<.0001
	Age x Segment	0.007	0.000	0.000

Relative expression values, expressed as arbitrary units, are presented as LSMeans with (SEM) with n = 5 to 7 per experimental group.

a–f Means with different superscripts within the same column are significantly different (*P* < 0.05).

*VDR* = Vitamin D receptor; *SLC20A1* = Solute carrier family 20 member 1; *SLC34A2* = Solute carrier family 34 member 2.

## DISCUSSION

**Figure 3.**
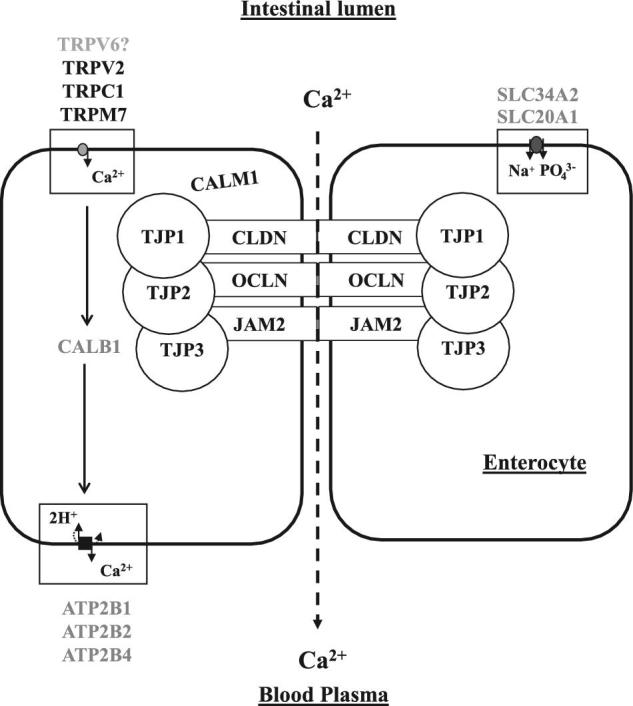
Hypothetical model of ionic calcium (Ca) and phosphate (P) transfer across the intestinal epithelial barrier of the laying hen. Rectangles represent the transmembrane proteins, junctional adhesion molecule (JAM), claudins (CLDN: CLDN1, 2, 10, or 12), or occludin (OCLN), while the circles represent the tight junction proteins (TJP: TJP1, 2, or 3). Black arrows indicate the transcellular pathway and dotted arrows the paracellular pathway. Genes in grey represent those previously identified in the small intestine of laying hens, while genes in black were those identified in the present study. Ca^2+^ refers to ionic calcium, PO_4_^3−^ to the phosphate ion, H^+^ to the proton ion and Na^+^ to the sodium ion. TRPV6 = Transient receptor potential cation channel subfamily V member 6; TRPV2 = Transient receptor potential cation channel subfamily V member 2; TRPC1 = Transient receptor potential cation channel subfamily C member 1; TRPM7 = Transient receptor potential cation channel subfamily M member 7; CALM1 = Calmodulin; CALB1 = Calbindin 28K; ATP2B1 = ATPase plasma membrane Ca^2+^ transporting 1; ATP2B2 = ATPase plasma membrane Ca^2+^ transporting 2; ATP2B4 = ATPase plasma membrane Ca^2+^ transporting 4; CLDN2 = Claudin 2; CLDN12 = Claudin 12; CLDN10 = Claudin 10; CLDN1 = Claudin 1; OCLN = Occludin; JAM2 = Junctional adhesion molecule 2; TJP1 = Tight junction protein 1; TJP2 = Tight junction protein 2; TJP3 = Tight junction protein 3; VDR = Vitamin D receptor; SLC20A1 = Solute carrier family 20 member 1; and SLC34A2 = Solute carrier family 34 member 2.

In the present study, RT-qPCR was used to quantify mRNA expression of candidate genes of the transcellular and the paracellular Ca absorption pathways in the small intestine of laying hens. An integrative description of genes involved in Ca and P absorption in mature laying hens is provided in the Figure 3. Concerning the transcellular pathway, we identified three additional candidate genes for Ca^2+^ entry. We also confirmed that *CALB1* increased in the duodenum of mature laying hens, and observed that it also increased in the jejunum. Two candidate genes for Ca extrusion *ATP2B1* and *ATP2B2* rose respectively, in all 3 segments or in the duodenum of laying hens. This study also detected the expression of candidate genes of the paracellular pathway for the first time. Gene expression of *CLDN2, CLDN12, TJP1, TJP2*, and *TJP3* increased with age or only after SM, or in 1 or 2 segments of the small intestine. The up regulation of *CLDN2* and *CLDN12*, which increase Ca^2+^ permeability in intestinal epithelial cells, supports the hypothesis that the paracellular pathway may be involved in Ca absorption in laying hens.

Sexual maturity in birds is a period of profound physiological modifications, linked to the onset of the reproductive function and marked by the first oviposition (García-Fernández et al., 2015). In layer production units, this transition period requires appropriate environmental conditions and adequate nutrition, which were respected in the present study (Dawson et al., 2001). The activity of the gonadotropic axis results in estrogen synthesis by the ovary which in turn promotes the development and growth of various target organs, like the oviduct (Pearce, 1974; Johnson, 2000). In the present study, although steroids were not assayed, the increase of oviduct weight between 12 and 17 wk old was the hallmark of their effects (Munro and Kosin, 1943; Dougherty and Sanders, 2005). Plasma parameters were modified during this transition period from immature to mature hens. The huge rise in total plasma Ca observed after SM is consistent with previous observations. It results from the feed transition with higher dietary Ca levels (from 1 to 3.5%) and increased vitellogenin synthesis, an egg yolk precursor containing binding sites for Ca (Nys et al., 2011). A decrease in total plasma P was observed at 17 wk old, when medullary bone was first detected, and coincided with the increase of 1.25(OH)_2_D_3_, previously described by Castillo et al. (1979). We also observed a decrease in 25(OH)D_3_ at 17 wk old, in contrast to a previous study (Nys et al., 1986a). This might reflect a limiting activity of the enzyme converting dietary cholecalciferol to 25(OH)D_3_ (25-hydroxylase), rather than a limiting plasma level of vitamin D binding protein, or insufficient vitamin D dietary supply high at this stage (Nys et al., 1986a). In the present study, *CALB1* mRNA expression was enhanced in the duodenum after SM, as observed previously for CALB1 protein and mRNA levels, which correlated with the increased Ca absorption capacity in laying hens (Nys et al., 1986b, 1992). These observations indicate that the present experiment is particularly suited to the identification of new candidate genes of the transcellular and paracellular Ca absorption pathways in laying hens.

The present experiment provides the expression profile of 7 candidate genes of the transcellular Ca transport in addition to *CALB1*, in the small intestine of pullets at several ages and of laying hens, for the first time. The intestinal transcellular pathway occurs in 3 steps: apical Ca^2+^ entry, intracellular Ca^2+^ transport and basal active Ca^2+^ extrusion (Wasserman and Taylor, 1966; Coty and Conkey, 1982). We did not detect *TRPV6* in the current study at any age, although previous studies have reported its expression in the intestine of the laying hen (Yang et al., 2011; Jonchère et al., 2012; Li et al., 2018). This observation is in agreement with the absence of a *TRPV6* expression in the chicken RNAseq data and in growing broiler chickens, despite the detection of an immunoreactive TRPV6 protein in the chicken's small intestine (Proszkowiec-Weglarz and Angel, 2013; Huber et al., 2015; Juanchich et al., 2018; Proszkowiec-Weglarz et al., 2019). Therefore, as previously discussed further studies are necessary to conclude about *TRPV6* expression in the hen's small intestine (Proszkowiec-Weglarz et al., 2019). Out of the 4 candidate genes for apical Ca^2+^ entry studied, 3 were detected. The first candidate, *TRPV2* is a Ca^2+^ channel (Owsianik et al., 2006). It varied neither with age, nor with the segment. *TRPM7* is a divalent cation channel (Chubanov et al., 2004; Owsianik et al., 2006). It was enhanced in the jejunum between 12 and 23 wk old. *TRPC1* is known to facilitate Ca^2+^ entry (Ambudkar et al., 2007). Its expression increased in all 3 segments between 12 and 15 wk old. We hypothesize that these 2 new candidates (*TRPM7* and *TRPC1)* could compensate for the absence of *TRPV6* expression in the intestine of laying hens. *CALM1* is a ubiquitous Ca^2+^ binding protein, acting as an inhibitor of TRP channel activities (Zheng, 2013). It did not vary consistently between segments or ages. Although none of the candidates for Ca entry was expressed differentially between 17 and 23 wk old, this does not exclude variations at protein level or activity.

The high absorption of Ca in the small intestine of laying hens implies mechanisms for maintaining a low free intracellular concentration of Ca^2+^ (Carafoli, 1987). It has been proposed that *CALB1*, discovered in chick intestinal epithelial cells, acts as a buffer through intracellular Ca^2+^ binding (Wasserman and Taylor, 1966). The increased expression of *CALB1* previously described in the duodenum after SM, is extended to the jejunum in the present study, which further highlights the importance of this candidate in the saturable Ca transport in these two segments (Nys et al., 1992).

The output of Ca^2+^ from the enterocyte occurs against a concentration gradient and involves plasma membrane Ca^2+^ ATPases (Bar, 2009). Four isoforms have been characterized in mammals (*ATP2B*1, *B2, B3* and *B4*), but only 3 are maintained in birds *ATP2B1, B2*, and *B4* and these have already been detected in the hen's duodenum (Strehler and Zacharias, 2001; Jonchère et al., 2012). On the one hand, the results may indicate that *ATP2B1, ATP2B2*, and *CALB1* are favored partners of the transcellular Ca absorption pathway in the duodenum of laying hens, where they are highly expressed. On the other hand, *ATP2B4* could be a favored partner to mediate Ca^2+^ output in the ileum of the laying hens, where it is highly expressed, and could compensate for the comparatively lower expression of *ATP2B1* and *ATP2B2*. Due to the higher pH in the ileum, which lowers Ca solubility, its absorption rate is lower than the upper segments (Hurwitz and Bar, 1968). It remains, however the high expression of *ATP2B4* combined with a low expression of *CALB1* could contribute to this difference. Although several studies and reviews proposed that non-saturable transport could contribute to intestinal Ca absorption in birds, candidate genes of the paracellular pathway have been given little attention so far (Hurwitz and Bar, 1969; Nys and Mongin, 1982). The TJ are the functional units of this pathway, composed of transmembrane proteins CLDN, JAM, OCLN, and cytosolic proteins TJP, forming pores for the permeation of ions and water. The expression data showed for the first time, that all candidates were expressed in the hens’ small intestine, supporting the hypothesis that this pathway could contribute to Ca transfer in the laying hens’ intestine (Hurwitz and Bar, 1969; Nys and Mongin, 1982). *CLDN1* decreases cation permeability (Gunzel and Yu, 2013). It did not show any variation in this experiment. *CLDN2* and *CLDN12* are known to increase intestinal Ca^2+^ permeability (Fujita et al., 2008). In the present trial, CLDN*12* expression was the highest in the jejunum across all ages studied, whereas *CLDN2* only appeared after SM in this segment. In the duodenum, *CLDN2* expression increased with age. Both segments support the increase of intestinal Ca absorption during shell formation, when high contents of soluble Ca are present in the intestine (Hurwitz and Bar, 1965, 1969; Sauveur and Mongin, 1983; Guinotte et al., 1995). These segments are the main Ca absorption sites, in contrast to the ileum, and their high rate of Ca absorption may largely result from a non-saturable Ca transport, because CALB1 concentration does not vary during the daily ovulatory cycle (Hurwitz and Bar, 1966, 1968; Nys et al., 1992). In contrast, *CLDN10* decreased with age across all 3 segments. Two splice variants of *CLDN10* were initially described in mice with opposite charge selectivity, and 4 additional ones were reported (Van Itallie and Anderson, 2006; Gunzel et al., 2009). At least 2 splice variants exist in *Gallus gallus* which cannot be distinguished in the present study. The reduction in *CLDN10* expression with age may suggest a role that is distinct from that of *CLDN2.* Although the precise role of TJP proteins for Ca transport is unknown, these proteins anchor the TJ (Suzuki, 2013). The candidate *TJP2* was most expressed in the jejunum and *TJP3* in the ileum. All three *TJPs* were up-regulated after SM, suggesting their importance in laying hens. The expression of *JAM2* and *OCLN* remained at similar levels between intestinal segments after SM. Although these two candidate genes contribute to overall intestinal permeability, further research is needed to understand their role in Ca absorption (Laukoetter et al., 2007). Based on their regulation pattern across ages and segments, *CLDN2, CLDN12, TJP1, TJP2*, and *TJP3* are strong candidate genes, which may contribute to paracellular Ca absorption in the intestine of the sexually mature hen.

Phosphorus and Ca metabolisms are tightly linked and regulated by the same hormones (Kuro-o and Moe, 2017). An optimal absorption of P is crucial during the formation of the medullary bone before the onset of laying and later during the daily ovulatory cycle when an intense bone remodeling occurs (Castillo et al., 1979; Sauveur and Mongin, 1983; Kerschnitzki et al., 2014). In the jejunum, the expression of *SLC20A1* decreased, but that of *SLC34A2* increased with age. These results pointed to *SLC20A1* as the main P transporter in the ileum, and *SLC34A2* as the main transporter in the duodenum and jejunum after SM. However, the jejunum exhibited an intermediate level of expression of both transporters which could contribute to the relatively high absorption of P in this segment (Hurwitz and Bar, 1965).

The nuclear *VDR* was expressed in all 3 parts of the small intestine and at similar levels across ages. The increase in 1.25(OH)_2_D3 with age and after SM triggers the expression of target genes in the small intestine by activating its receptor (Dusso et al., 2005; Nys and Le Roy, 2017). Gene expression of *CALB1* increased after SM, when 1.25(OH)_2_D_3_ rose sharply. This is consistent with the presence of a vitamin D responsive element on the gene sequence of *CALB1* (Dusso et al., 2005; Nys and Le Roy, 2017). Several other genes, which were induced (*TRPM7, TRPC1, ATP2B1, ATP2B2, ATP2B4, CLDN2, TJP1, TJP2, TJP3*, and *SLC34A2*) or repressed (*ATP2B4, CLDN10, JAM2*, and *SLC20A1*) with age or after SM, could also be regulated by vitamin D. Some have already been shown to be responsive to vitamin D, like *ATP2B1, SLC34A2, CLDN2*, and *CLDN12* (Cai et al., 1993; Fujita et al., 2008; Forster et al., 2013).

In birds, most dietary Ca is absorbed in the upper parts of the small intestine (the duodenum and jejunum), while the main site of absorption in mammals is the ileum (Bar, 2009). This could be attributed to the comparatively shorter length of the entire small intestine, the faster passage rates and the intrinsic Ca absorption rates of the different compartments in birds (Hurwitz and Bar, 1966, 1968; Caviedes-Vidal et al., 2007). The present study suggests that molecular mechanisms underlie these differences. Indeed *CLDN2, CLDN12, CALB1, ATP2B1*, and *ATP2B2* were enhanced after SM in the duodenum or jejunum. The transcellular pathway cannot be sufficient to support Ca absorption in laying hens, as it is saturable. Our results highlight that 4 candidates of the paracellular pathway (*CLDN2, TJP1, TJP2*, and *TJP3*), as well as 3 candidates of the transcellular pathway (*CALB1, ATP2B1*, and *ATP2B2*) are enhanced after SM in the duodenum or the jejunum or the 3 intestinal segments. Therefore, we believe that both pathways work cooperatively to support the high rate of Ca absorption, when a high content of soluble Ca is present in the intestine during eggshell formation (Hurwitz and Bar, 1968; Sauveur and Mongin, 1983; Hwang et al., 2013). It would therefore be interesting to sustain higher intestinal levels of soluble Ca during the last third of eggshell formation. This would increase the non-saturable transport of Ca, and thereby Ca retention, a pre-requisite for reducing bone mobilization and P losses. Understanding the regulation of candidate genes of both pathways by 1.25(OH)_2_D_3_ could also be of interest in older hens, as a deficiency in the vitamin D metabolite conversion was suggested (Abe et al., 1982; Frost and Roland, 1990). This deficiency could possibly be alleviated by enhancing the expression of genes involved in both Ca absorption pathways, through a dietary supply of vitamin D_3_ metabolites.

To conclude, this study identified 17 candidate genes of the transcellular and paracellular Ca transport pathways in the 3 distinct parts of the laying hen's small intestine at different ages, before and after SM, and suggested the importance of the paracellular pathway in Ca absorption. The present expression data sets the basis for future studies providing the final proof of their physiological relevance, though the detection of the corresponding proteins and the measure of their activity. Both transcellular and paracellular pathways may work cooperatively in the duodenum and the jejunum, the main sites of Ca absorption in laying hens. The non-saturable transport through the paracellular Ca pathway could be a mechanism of great importance in laying hens due to the high amount of soluble Ca in the intestine. The results also provide new insights to explain the differences in Ca absorption rates between the duodenum, jejunum and ileum in laying hens, through adaptations of the molecular repertoire of Ca transporters.
